# The outcomes of the most severe polytrauma patients: a systematic review of the use of high ISS cutoffs for performance measurement

**DOI:** 10.1007/s00068-023-02409-3

**Published:** 2023-12-18

**Authors:** Benjamin M. Hardy, Adrian Varghese, Megan J. Adams, Natalie Enninghorst, Zsolt J. Balogh

**Affiliations:** 1 Department of Traumatology, John Hunter Hospital and University of Newcastle, Newcastle, NSW 2310 Australia; 2 University of Newcastle, Newcastle, NSW Australia; 3 Injury and Trauma Research Program, Hunter Medical Research Institute, Newcastle, Australia

**Keywords:** Polytrauma, Trauma, Multiple trauma, Trauma centre, Trauma care, Trauma surgery

## Abstract

**Background:**

This systematic review aimed to describe the outcomes of the most severely injured polytrauma patients and identify the consistent Injury Severity Score based definition of utilised for their definition. This could provide a global standard for trauma system benchmarking.

**Methods:**

The Preferred Reporting Items for Systematic Reviews and Meta-Analyses (PRISMA) checklist was applied to this review. We searched Medline, Embase, Cochrane Reviews, CINAHL, CENTRAL from inception until July 2022. Case reports were excluded. Studies in all languages that reported the outcomes of adult and paediatric patients with an ISS 40 and above were included. Abstracts were screened by two authors and ties adjudicated by the senior author.

**Results:**

7500 abstracts were screened after excluding 13 duplicates. 56 Full texts were reviewed and 37 were excluded. Reported ISS groups varied widely between the years 1986 and 2022. ISS groups reported ranged from 40–75 up to 51–75. Mortality varied between 27 and 100%. The numbers of patients in the highest ISS group ranged between 15 and 1451.

**Conclusions:**

There are very few critically injured patients reported during the last 48 years. The most critically injured polytrauma patients still have at least a 50% risk of death. There is no consistent inclusion and exclusion criteria for this high-risk cohort. The current approach to reporting is not suitable for monitoring the epidemiology and outcomes of the critically injured polytrauma patients.

**Level of evidence:**

Level 4—systematic review of level 4 studies.

**Supplementary Information:**

The online version contains supplementary material available at 10.1007/s00068-023-02409-3.

## Introduction

Trauma is a disease from energy transfer to the organism [1]. Above a certain threshold of energy deposition, the human body is unable to dissipate energy rapidly enough and physical injury results. The spectrum of injury runs from trivial superficial injuries to unsurvivable complete destruction of the body. Major trauma remains a persistent threat to life and function [2]. Advances in injury prevention and trauma care throughout the twentieth century, and particularly the last 20 years, have reduced the risk of dying from injury in most of the high-income countries of the world [3]. To further study and benchmark, trauma surgeons have sought to classify trauma patients by severity to measure performance and allow comparison between systems [4]. Classification and benchmarking systems must use common thresholds and cutoffs to allow comparison between centres and time periods.

For practical reasons, groups compared are limited by a minimum injury severity above which they are referred to as major trauma or severely injured. To allow fair comparison, this threshold requires a standardized scoring system that is valid across continents and populations. The most frequently used anatomical scoring system, the Injury Severity Score (ISS) is derived from the Abbreviated Injury Scale (AIS) [5]. The AIS a highly detailed scoring system developed to describe and rank injury severity (from 1 to 6) across the human body [6]. The AIS requires skilled data coders but as a purely anatomic system allows quality assurance and in-hours coding from the medical record. The ISS is a scale from 1 to 75 and is derived from the sum of the squares of the three highest AIS body regions [5]. The most commonly used ISS threshold is ≥ 16 [7]. This threshold is the inflection point between ISS 14 and 16 where trauma mortality began to exceed 10%. Below this the risk of death falls while also including the increasingly large numbers of less severely injured patients. Even above ISS ≥ 16 the distribution of severity of injuries is skewed towards the mild end of the spectrum of disease. For example in the New South Wales (Australia) Trauma Registry, 62% of patients have an ISS < 25 and 96% of patients have an ISS < 41 [8]. An increase in the proportion of patients with lower injury severity, or an expansion of the definition of major trauma to include less severely injured patients would further dilute the influence that the most severely injured patients would have on summary statistics used to characterize and compare the outcomes and performance of major trauma care. If the threshold for ‘major trauma’ is expanded to ISS ≥ 13, the inclusion of these additional low ISS patients further dilutes the high ISS patients. An expansion of the definition of major trauma to this level would expand the proportion of patients with an ISS of < 25–72% and those having an ISS of < 41–97%.

In its original description, the ISS was not categorized into groups. There are a variety of injury severity thresholds initially proposed at geometric nexuses in the calculation of the ISS from its basis in AIS. Debate exists surrounds the floor threshold defining injury severity given variability in some AIS injury classification updates with proposals to reduce the floor to ISS > 12 [9]. ISS subgroups were first proposed in the initial description of ISS noting that “scores below 10 rarely die” [5]. ISS 50–75 was first described as a group in 1988 [10]. In 1990, the Major Trauma Outcome Study, a pivotal study in the development of measurement and risk adjustment of trauma mortality, referred to ISS 50–74 and separated ISS 75 [11]. ISS 50–75 has been used intermittently and with no standardized term terminology, variably and perhaps accidentally including ISS 48 due to discrepancies between > and ≥ . In 1999, ISS 50–75 was still referred to as patients with “fatal injuries” despite its patients having a mortality of approximately 50% [12]. In 2015 a major binational study confirmed it as a repeatable, useful ‘most severely injured’ population balancing patient numbers with comparable mortality from the triplets of included injuries across multiple nations [13].

Beyond mortality, the outcomes of the ISS 50–75 group remain unknown. It is also unknown whether the improvements in trauma mortality over time have improved the mortality in the most severely injured, ISS 50–75 group or whether this remains stubbornly high [14]. No major trauma registry currently benchmarks functional outcomes between centres [15].

The systematic review aimed to describe the outcomes of the most severely injured polytrauma patients and demonstrate the possible incomplete and varied application of injury severity subgroups at the most severely injured end of the spectrum of major trauma.

## Methods

### Selection criteria and search strategy

Studies were included if they used a floor ISS threshold of 40 or above and measured outcomes of mortality. Medline, Embase, Cochrane Reviews, CINAHL, CENTRAL were searched using a variety of search terms including “’ISS, ‘injury severity’, ‘died’, ‘mortality’, ‘outcome’, ‘severe’” (SDC1). Published studies up to July 2022 were included.

### Study selection, and inclusion and exclusion criteria

Review and extraction were conducted in Covidence (Covidence systematic review software, Veritas Health Innovation, Melbourne, Australia. Available at www.covidence.org). All abstracts were reviewed by two reviewers with ties broken by the senior author. Studies were included if they summarized the outcome of patients after major injury. All full texts were reviewed by the first author and senior author, and ties resolved by consensus. Studies were excluded if there was no separation of patients into ISS categories, if the maximum ISS category was not ≥ 40, or the study was duplicated. Data was extracted by the first author and summarized in table form.

### Study quality and risk of bias

Studies’ inclusion and exclusion criteria were captured and are presented to the reader. The PRISMA 2020 checklist was used when designing and drafting the manuscript [16] (SDC 2).

## Results

**Fig. 1:**
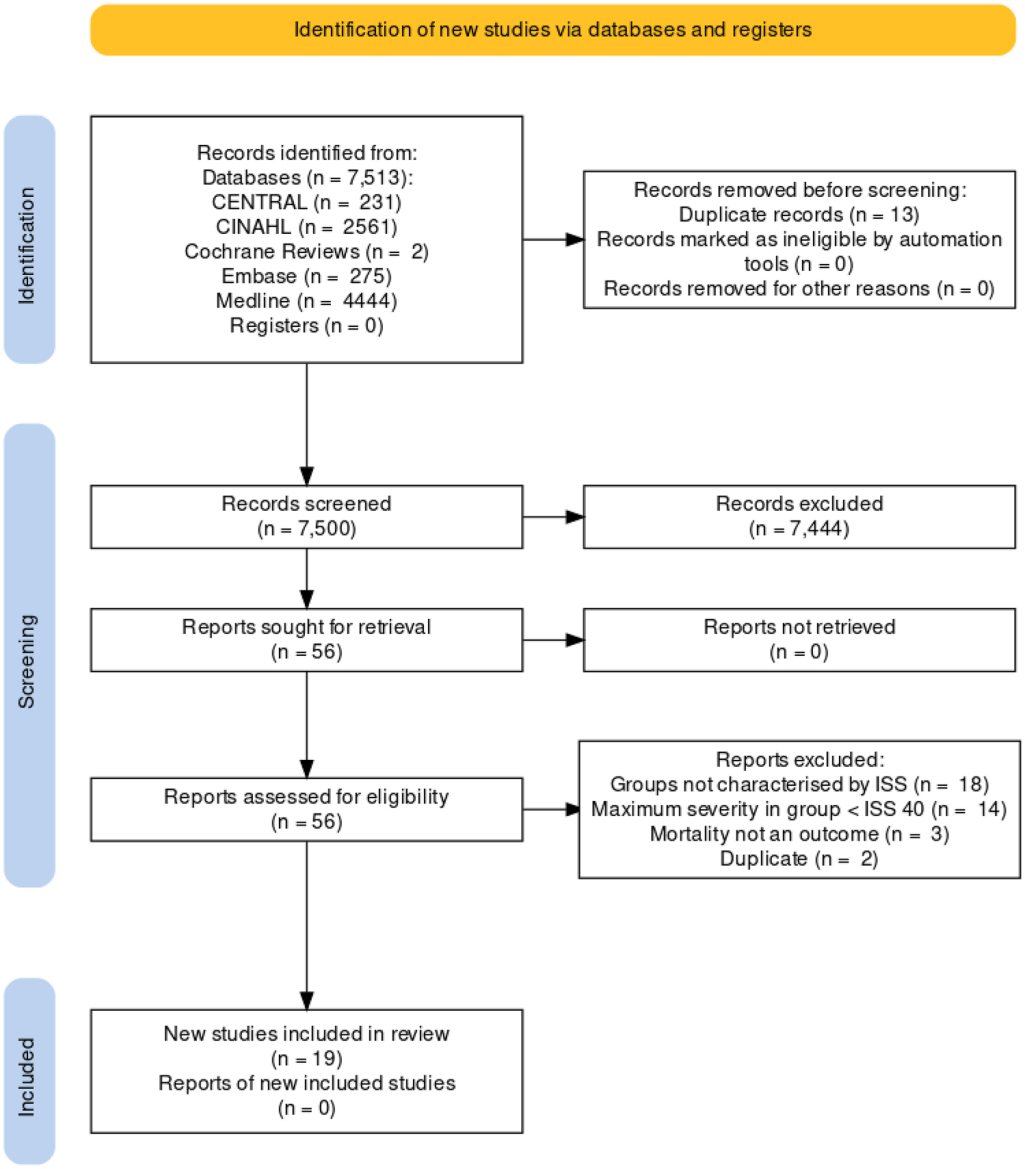
PRISMA flow diagram

Up to July 2022, 7,513 studies were identified with 13 duplicates, leaving 7500 studies. All abstracts were reviewed and 56 identified for full text screening. 37 were excluded, primarily due to no separation of patients into ISS categories (Fig. 1). 19 studies were included for final extraction [12, 13, 17–33] (Fig. 1, Table 1).

**Table 1:** Summary of included studies

Study ID	Country	Aim of study	Study design	Start date	End date	Population description	Inclusion criteria	Exclusion criteria	Total number of participants	ISS/NISS	Max severity group	Number of patients in max severity	Mortality % for above group
vanderSluis 1996 [31]	Nederlands	Compare mortality of young (20 to 29 years) and elderly (greater than or equal to 60 years). **Younger**	Retrospective	1/01/1985	1/01/1990	Injured patients	ISS > 15, Aged either 20–29 or ≥ 60	Not defined	161	ISS	50 +	unknown	50%
vanderSluis 1996 [31]	Nederlands	Compare mortality of young [13, 20] and elderly (greater than or equal to 60 years). **Older**							121	ISS	50 +	unknown	100%
Kaweski 1990 [26]	United States	Measure effect of fluid resus	Database	1/01/1985	31/07/1987	Injured patients	Not defined	Not defined	6855	ISS	> 50	190	84.1%
Sampalis 1999 [12]	Canada	Performance measurement	Prospective	1/04/1993	31/03/1998	Injured patients	Died, admitted, ISS > 13, PHI > 3, 2xAIS > 2 injuries, LOS > 3	Prehospital deaths	12,208	ISS	50 +	1032	75.3%
Burdett-Smith 1995 [19]	UK	Before-after study. Trauma team and audit implementation development. **Before**	Retrospective	1/10/1988	1/04/1992	Injured patients	ISS > 15	Not defined	186	ISS	> 41	34	91%
Burdett-Smith 1995 [19]	UK	Before-after study. Trauma team and audit implementation development. **After**							198	ISS	> 41	41	76%
Sampalis 1995 [30]	Canada	Compare ISS-defined mortality risk with expert consensus	Retrospective	1/04/1987	31/4/1988	Injured patients	Died, admitted	Not defined	116	ISS	50 +	18	83.3%
Kuhne 2005 [27]	Germany	Identify risks for mortality	Database	1/01/1993	31/12/2000	Injured patients	ISS 16 + , Age 15 +	Not defined	5375	ISS	> 50	289	66.1%
Russell 2004 [29]	Australia	Test differences in mortality between ISS-equivalent AIS triplets	Database	1/01/1995	23/06/1995	Injured patients	Not defined	AIS 1 or 2 to single region	5946	ISS	50 +	44	77.2%
Jamulitrat 2001 [25]	Thailand	Compare ISS and NISS at predicting mortality	Prospective	1/06/1996	31/05/1999	Injured patients	Not defined	Not defined	2044	Both ISS and NISS	46 +	15	80%
Fatovich 2013 [24]	Australia	Identify risks for mortality	Database	1/07/1997	30/06/2006	Injured patients	Admission > 24 h or death	Not defined	3214	ISS	50 +	239	57.3%
Vyhnanek 2012 [32]	Czech Republic	Performance measurement	Retrospective	1/01/2009	31/12/2010	Injured patients	Admission	Not defined	515	ISS	40 +	95	65%
Wurm 2012 [33]	Germany	Characterise long-term outcome of ISS50 + patients	Retrospective	1/01/2000	1/12/2005	Injured patients	ISS > 15	Not defined	1435	ISS	50 +	88	36.4%
Rozenfeld 2014 [13]	Israel	Define useful ISS groups	Database	1/01/1998	31/12/2011	Injured patients	All trauma patients	Isolated hip fractures, no ISS calculated	566,094	ISS	50 +	1451	58%
Bagher 2015 [17]	Sweden	Identify risks for mortality	Retrospective	1/01/2011	31/12/2013	Injured patients	Trauma team activation	Prehospital deaths, burns, drowning, asphyxia	428	NISS	40 +	31	67.7%
Ball 2015 [18]	Canada	Epidemiology	Database	1/01/1995	31/12/2011	Injured patients	Age ≥ 16 year, ISS ≥ 12	Not defined	12,879	ISS	48 +	Not listed	27%
Duvall 2015 [23]	United States	Predict futile care	Database	1/01/2007	31/12/2012	Injured patients	NTDB inclusion criteria	ISS 75	570,442	ISS	50–59, 60 +	828 + 48 = 876	ISS 50–59 = 66.9%, ISS 60 + = 79.1% Overall = 592/876 = 67.6%
Mann 2018 [28]	Canada	Epidemiology	Retrospective	1/04/2005	31/03/2015	Pelvic fracture patients	Pelvic fractures	Patients with low energy mechanisms, and an Injury Severity Score (ISS) of < 16	3915	ISS	50 +	401	37.4%
Cameron 2020 [20]	Australia	Epidemiology	Database	1/07/2016	30/06/2017	Injured patients	ISS > 12 or death	Delayed presentation > 7d, poisoning, foreign bodies, isolated NOF#, patients with delayed admission, " older adults (> 64 years of age) who died with superficial injuries only"	8423	ISS	41 +	302	44.0%
Candefjord 2020 [21]	Sweden	Compare trauma and non-trauma centre care	Database	1/01/2013	31/12/2017	Injured patients	Trauma team activation, NISS > 15	Missing data, ISS not calculated	44,984	ISS	50 +	242	69.8%

### Groups used and study types

The minimum threshold for inclusion in the ‘most severe’ group was not consistent and included ≥ 40, > 41, ≥ 46, ≥ 48, ≥ 50, 50–59, > 50, and ≥ 60. Some studies relied on prospective registry collection with the rest retrospective. There were two studies with predefined aims and prospective collection. Most studies’ aims were epidemiological. One study sought to measure the effect of prehospital treatment. Others tested change over time either in before-and-after or year-on-year designs during the development of trauma care systems. One study compared outcomes of patients treated in trauma-centres with against those treated in non-trauma centres.

### Study quality

There was variable reporting of population, inclusion, and exclusion criteria. Few studies reported exclusion of prehospital death and no studies reported the use of autopsy to inform ISS score generation in prehospital deaths.

### Outcomes

**Fig. 2:**
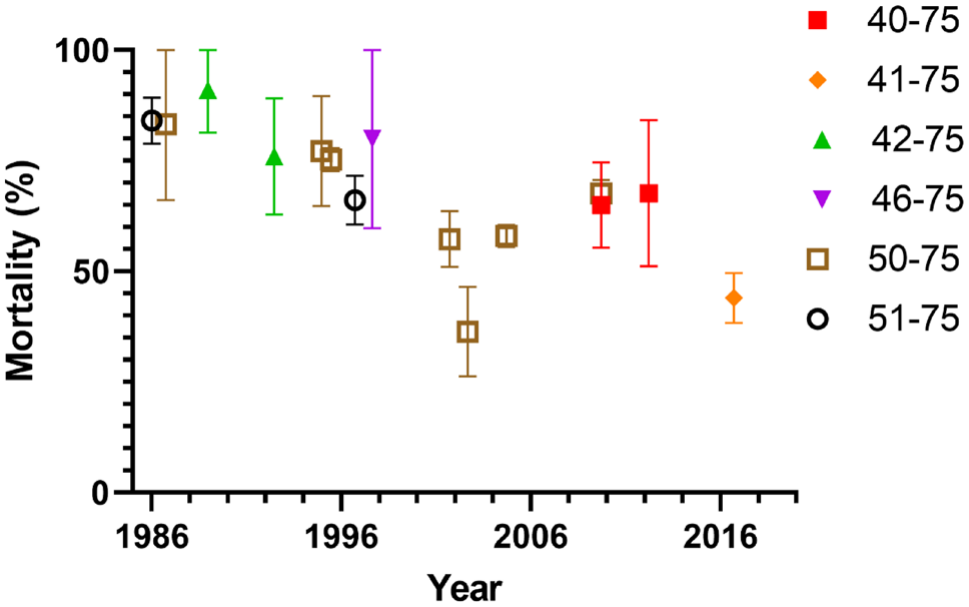
Change in mortality over time. Error bars are calculated 95% confidence intervals from listed sample sizes. Studies not plotted either did not list sample sizes [31] and did not respond to requests for additional data [18], or reported a subpopulation (e.g., pelvic fracture) only [28]

Mortality of ‘most severe’ groups with an ‘all-comers’ inclusion criteria ranged from 27% [18] to 91% [19] (Fig. 2).

## Discussion

Our systematic review demonstrates the limited and incomplete use of a ‘most severely injured’ group in trauma outcome reporting. A variety of high ISS cutoffs have been used in the literature. Most are used without citation or justification. Rozenfeld et al. demonstrated that 50–75 is the most functional high ISS group and remains the largest series to date [13]. Russel et al. demonstrated the considerable variability in mortality rate of component AIS triplets at the same ISS level at most levels below an ISS of 50, advancing its use over lower cutoffs such as ≥ 40 as is used in the NSW Trauma Registry [8, 29, 34].

Our review is strong in that it had no limitations in calendar year or language and use a broad search criterion with manual review of 7,500 titles and abstracts. It is weakened by the lack of standardized reporting language around high ISS groups.

In conclusion, there is considerable variation in the definition and reporting of the ‘most severe’ group of trauma patients. The outcomes of these patients are uncertain but include at least a 50% risk of death. Authors should standardize on ISS50-75 given its large, well validated measure of the most severely injured [13]. Major registries should adopt the ISS50-75 group as a public performance measure to educate other authors in its standardization. International consensus efforts regarding high ISS groups should standardize on language to reduce the burden of search regarding high ISS groups such as ISS > XX or ISSXX-YY (ISS > 48, ISS ≥ 50, ISS50-75). Editors could continue to encourage authors to standardize terms used to describe injury severity groups within polytraumatized patients.

## Supplementary Information

Below is the link to the electronic supplementary material.

Supplementary file1 (DOCX 19 kb)

Supplementary file2 (DOCX 30 kb)
