# Supplementary material for: The outcomes of the most severe polytrauma patients: a systematic review of the use of high ISS cutoffs for performance measurement
Source: Eur J Trauma Emerg Surg. 2023 Dec 18;50(4):1305–12. doi: 10.1007/s00068-023-02409-3 (PMC11481685; doi:10.1007/s00068-023-02409-3)
Supplement: Supplementary file 1 — Supplementary file1 (DOCX 19 kb) [file 68_2023_2409_MOESM1_ESM.docx]

Appendix

Appendix 1 – High injury severity score systematic review search strategy

Searches conducted: 9^th^ September 2020

Update searches conducted 15^th^ July 2022

Searches saved in dfb777’s database profile –– Major trauma outcomes - <database name>

| Database | Vendor | Records identified | Records after deduplication |
| --- | --- | --- | --- |
| CENTRAL | Wiley | 369 | 201 |
| CINAHL | EBSCO | 3517 | 2162 |
| Cochrane Reviews | Wiley | 4 | 2 |
| Embase | OVID | 2850 | 218 |
| Medline | OVID | 4052 | 4037 |
| TOTAL |  | 10,792 | 6,620 |

Update searches – July 15, 2022

| Database | Vendor | Records identified | Records after deduplication |
| --- | --- | --- | --- |
| CENTRAL | Wiley | 50 | 30 |
| CINAHL | EBSCO | 632 | 399 |
| Cochrane Reviews | Wiley | 0 | 0 |
| Embase | OVID | 86 | 57 |
| Medline | OVID | 575 | 407 |
| TOTAL |  |  | 893 |

Database(s): Ovid MEDLINE(R) and Epub Ahead of Print, In-Process & Other Non-Indexed Citations and Daily 1946 to September 08, 2020
Search Strategy:

| # | Searches | Results |
| --- | --- | --- |
| 1 | Injury Severity Score/ | 16034 |
| 2 | (iss or injury severity score*).tw. | 15024 |
| 3 | 1 or 2 | 26016 |
| 4 | exp "Outcome and Process Assessment, Health Care"/ | 1167768 |
| 5 | outcome*.tw. | 1747955 |
| 6 | mortality/ or (death* or died or mortalit*).tw. | 1577152 |
| 7 | 4 or 5 or 6 | 3627605 |
| 8 | "Wounds and Injuries"/cl [Classification] | 2482 |
| 9 | ((major* or sever* or critical*) adj (injur* or trauma*)).tw. | 25553 |
| 10 | 8 or 9 | 27680 |
| 11 | 3 and 7 and 10 | 4052 |

Database(s): **Embase**1947 to present
Search Strategy:

| # | Searches | Results |
| --- | --- | --- |
| 1 | (iss or injury severity score*).mp. | 23773 |
| 2 | clinical outcome/ | 142763 |
| 3 | outcome*.tw. | 2594758 |
| 4 | mortality/ or (death* or died or mortalit*).tw. | 2574064 |
| 5 | 2 or 3 or 4 | 4587083 |
| 6 | ((major* or sever* or critical*) adj (injur* or trauma*)).mp. | 35174 |
| 7 | 1 and 5 and 6 | 3667 |
| 8 | limit 7 to conference abstract | 817 |
| 9 | 7 not 8 | 2850 |

CINAHL Complete

| # | Query | Results |
| --- | --- | --- |
| S1 | (iss or "injury severity score*") | 5,828 |
| S2 | (MH "Outcomes (Health Care)") | 52,497 |
| S3 | TI outcome* OR AB outcome* | 643,651 |
| S4 | (MH "Mortality") | 29,399 |
| S5 | TI ( (death* or died or mortalit*) ) OR AB ( (death* or died or mortalit*) ) | 341,576 |
| S6 | S2 OR S3 OR S4 OR S5 | 917,338 |
| S7 | (MH "Wounds and Injuries/CL") | 1,066 |
| S8 | TI ( ((major* or sever* or critical*) n1 (injur* or trauma*)) ) OR AB ( ((major* or sever* or critical*) n1 (injur* or trauma*)) ) | 21,384 |
| S9 | S7 OR S8 | 22,238 |
| S10 | S1 AND S6 AND S9 | 3,517 |

COCHRANE LIBRARY

ID Search Hits

#1 MeSH descriptor: [Injury Severity Score] this term only 461

#2 (iss or "injury severity score*"):ti,ab 1089

#3 (OR #1-#2} 1453

#4 MeSH descriptor: [Outcome and Process Assessment, Health Care] explode all trees 146907

#5 outcome*:ti,ab 444311

#6 MeSH descriptor: [Mortality] this term only 542

#7 (death* or died or mortalit*):ti,ab 121235

#8 (OR #4-#7} 583344

#9 MeSH descriptor: [Wounds and Injuries] this term only and with qualifier(s): [classification - CL] 16

#10 ((major* or sever* or critical*) near/1 (injur* or trauma*)):ti,ab 2343

#11 (OR #9-#10} 2352

#12 (AND #3, #8, #11}
